# Trends in Use of Direct‐Acting Antivirals for Treatment of Hepatitis C Virus Infection in Australia 2016–2024

**DOI:** 10.1111/jvh.70082

**Published:** 2025-09-06

**Authors:** Chieu‐Hoang Ly Luong, Lisa Kalisch Ellett, Nicole Pratt, Kirsten Staff, Jack Janetzki

**Affiliations:** ^1^ Clinical and Health Sciences University of South Australia Adelaide South Australia Australia; ^2^ Quality Use of Medicines and Pharmacy Research Centre University of South Australia Adelaide South Australia Australia

**Keywords:** direct‐acting antivirals, dispensing trends, hepatitis C, market‐share, pan‐genotypic regimens

## Abstract

Direct‐acting antivirals (DAAs) have transformed hepatitis C virus (HCV) treatment in Australia since their inclusion on the Pharmaceutical Benefits Scheme (PBS) in 2016. Treatment has shifted from genotype‐specific to pan‐genotypic regimens, with glecaprevir/pibrentasvir and sofosbuvir/velpatasvir now recommended in clinical guidelines. This study examined trends in DAA dispensing in light of evolving treatment regimens. A retrospective analysis of publicly available PBS data was conducted, assessing monthly DAA dispensings from March 2016 to December 2024. Dispensings were summarised by count and proportion, PBS item code, schedule (general, private, or public hospital) and number of repeats as a proxy for treatment duration. Dispensing volumes of DAAs increased following PBS‐listing in March 2016, with the highest number of dispensings observed between 2016 and 2017 (average of 11,378 prescriptions dispensed per month). Dispensing rates subsequently declined, with an average of 1583 prescriptions dispensed per month from 2020 to 2024. Since introduction to market in August 2017, sofosbuvir with velpatasvir (pan‐genotypic regimen) has maintained an average market share of 55%. Glecaprevir/pibrentasvir (pan‐genotypic regimen) has maintained an average market share of 34% since its introduction in August 2018. Sofosbuvir/velpatasvir/voxilaprevir, listed on the PBS in April 2019, and used for salvage therapy, has had a smaller average market share of 4% since listing. Pan‐genotypic regimens now account for nearly all DAA use in Australia. Declining dispensing rates may reflect reduced new infections and treatment fatigue. Increasing retreatment rates underscore the need for ongoing monitoring and real‐world evaluations. Future head‐to‐head comparisons may support optimal regimen selection.

## 1 Introduction

Hepatitis C virus (HCV), a blood‐borne pathogen targeting the liver, is transmitted primarily via injection drug use, unsterile medical or body‐modification practices, and shared personal hygiene items [1] While a substantial proportion of infections remain asymptomatic, chronic HCV, defined as persistence beyond 6 months, develops in approximately 75%–85% of cases. In 2023, over 68,890 individuals in Australia were living with chronic HCV, frequently without symptoms [2]. If untreated, chronic HCV may progress to liver fibrosis, cirrhosis, hepatocellular carcinoma and ultimately liver failure [3].

The advent of direct‐acting antivirals (DAAs) brought a paradigm shift: with cure rates exceeding 95%, treatment regimens typically span 12 weeks and aim for sustained virological response (SVR)—undetectable HCV RNA 12 weeks post‐treatment. Australia began subsidising DAAs via the Pharmaceutical Benefits Scheme (PBS) in March 2016, aligning with WHO's 2030 HCV elimination target [3]. An estimated 43,360 of 230,000 Australians (~19%) initiated DAA therapy in the first year, with 88,790 treated by the end of 2020 [4, 5, 6]. Pan‐genotypic regimens (sofosbuvir/velpatasvir; glecaprevir/pibrentasvir) listed from August 2017 enabled simplified prescribing without genotyping and broader access across care settings [7, 8].

Notably, early DAA rollout addressed patients with advanced fibrosis and cirrhosis, while more recent therapy reflects a shift toward treating mild or moderate disease. Reinfection and retreatment demand remain significant, with 2023 figures indicating retreatment accounts for over half of cases and only 8% of those with HCV having received treatment [2, 3].

Notably, early DAA rollout addressed patients with advanced fibrosis and cirrhosis, while more recent therapy reflects a shift toward treating mild or moderate disease. Reinfection and retreatment demands remain significant, with 2023 figures indicating retreatment accounts for over half of cases and only 8% of those with HCV having received treatment [2, 3]. These patterns underscore persistent unmet needs in the Australian response.

The transformation in Hepatitis C treatment landscape seen in Australia is reflected and challenged globally. Worldwide DAA access accelerated between 2015 and 2016: initiations rose from approximately 1 million to 1.5 million people treated [9]. Nevertheless, treatment remains unevenly distributed: high‐income countries maintain consistently higher DAA uptake, North America and Europe lead in treatment volume per HCV case, while low‐ and middle‐income countries (LMICs) lag, both in access and uptake. These disparities stem in part from pricing, patent restrictions, and prescriber limitations (e.g., specialist‐only mandates), which disproportionately affect LMICs [10]. Substantial unmet needs persist, highlighting the importance of policy interventions to lower prices, enable generic production, and expand prescribing authority to reach elimination goals.

Given these dynamic shifts both locally and globally, and recognising evolving treatment patterns, the aim of this study is to describe dispensing trends of HCV therapies in Australia from March 2016 to December 2024, offering insight into DAA usage that supports national and global elimination strategies.

## 2 Methods

### 2.1 Context and Data Sources

**FIGURE 1:**
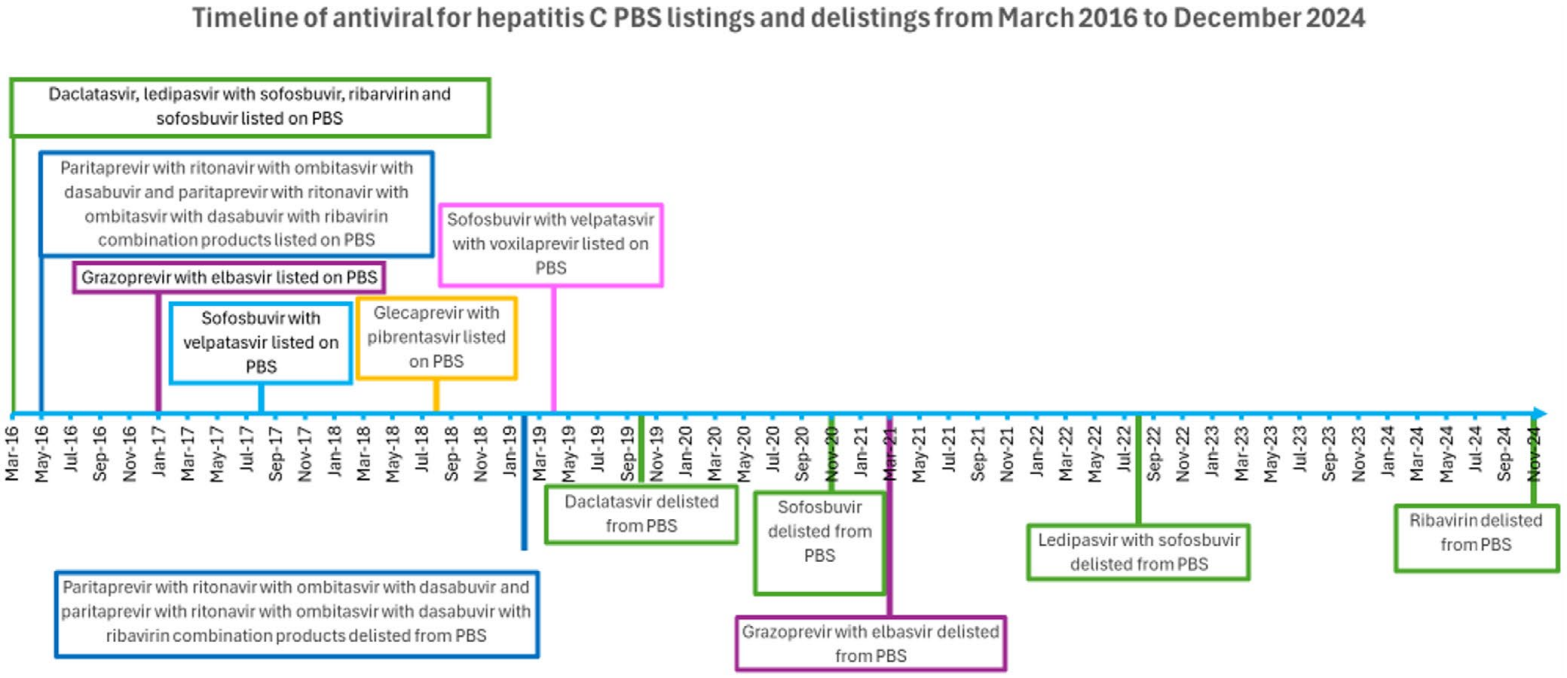
Timeline of Pharmaceutical Benefits Scheme listings and delisting's across study period.

In Australia, subsidised access to medicines is available to all residents via the national formulary, the Pharmaceutical Benefit Scheme (PBS). A timeline of PBS listing of DAAs for hepatitis C is provided in Figure 1. The Services Australia, Australian Government PBS Item Reports Statistics [11] was used to obtain counts of prescription dispensings processed for reimbursement claiming via the Pharmaceutical Benefit scheme from March 2016 to December 2024. This publicly available dataset includes aggregate monthly dispensings of each PBS item. Prescription medicines included in this study were all PBS listed medicines for Hepatitis C as identified by fourth level Anatomical Therapeutic Classification (ATC) code ‘J05AP’ [12]. The PBS item drug map [13] (January 2025 version) was used to select all J05AP medicines, their respective PBS item codes, medicine name, strength and formulation. A list of all medicines included in this analysis can be found in Table A1.

### 2.2 Statistical Reporting

We report claims of dispensings for HCV treatments by overall count and proportion per month, where the proportion is the percentage for each individual HCV treatment reported as a percentage of all HCV dispensings, between March 2016 and December 2024. Results are also reported according to whether they were dispensed in the General PBS schedule (as a proxy for medicines dispensed in the community setting), or on PBS Section 100 (as a proxy for medicines dispensed in the public or private hospital setting). General PBS Schedule refers to most medicines available on the PBS whereas Section 100 Highly Specialised Drugs (HSD) are medicines that require medical supervision and are usually for complex conditions. Both schemes are ways that the PBS subsidises medicines in Australia. The distinction between community and hospital use was made to allow for insight into where patients are obtaining dispensings. Results were also reported according to the maximum number of repeats allowed per PBS item, as a proxy measure of length of treatment. Although the data represent the number of claims for dispensing processed each month, PBS claiming for reimbursement is now completed in real time meaning that claim for payment dates closely resemble supply of medication dates. Therefore, we used the date of claims data as a proxy for the number of prescriptions dispensed each month and, for brevity, used this terminology in the reporting of results.

As this study used publicly available aggregate data, ethics approval was not required. Microsoft Excel Version 2408 was used for all analyses and visualisations.

## 3 Results

**FIGURE 2:**
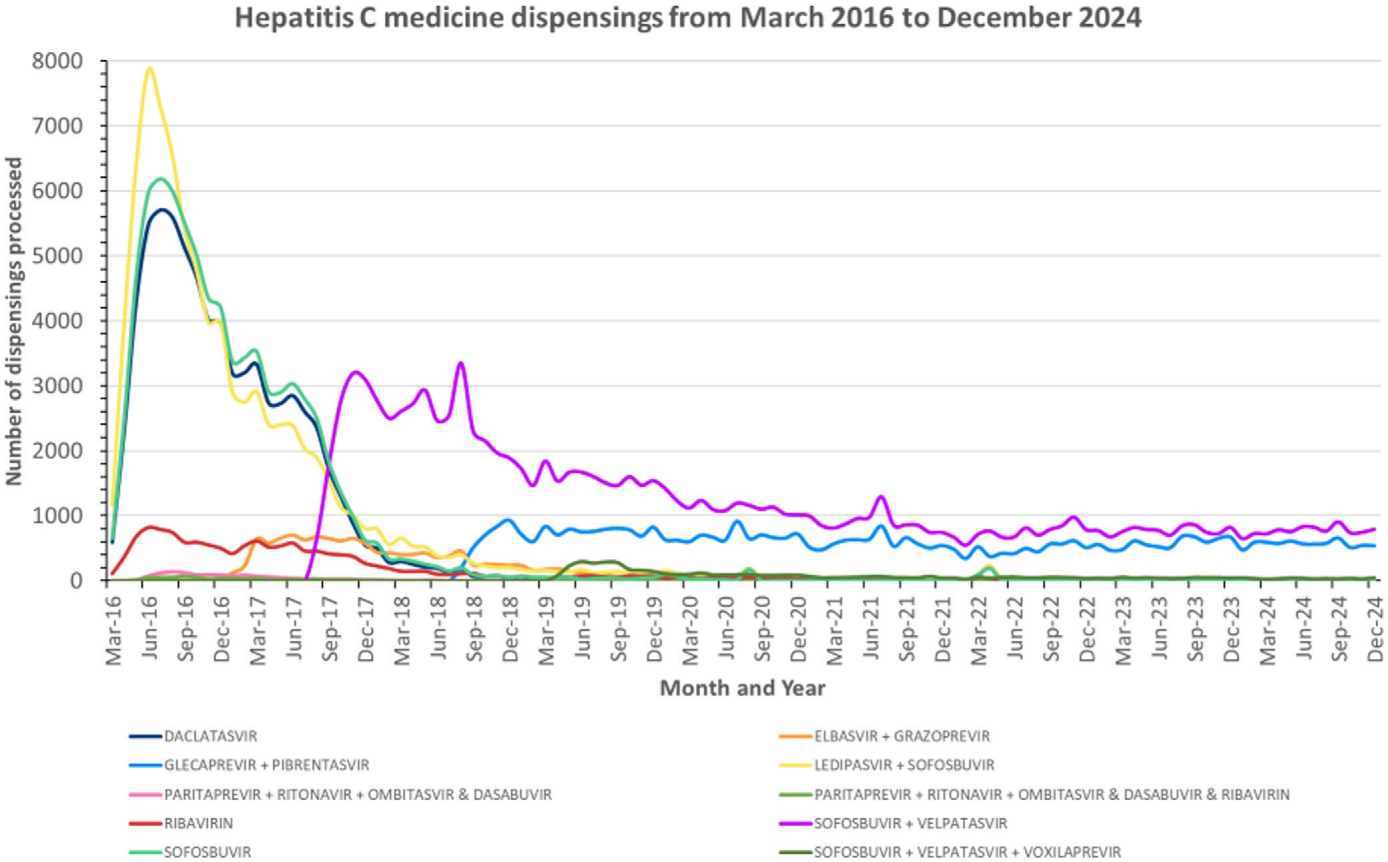
Monthly dispensing's of direct acting antivirals for hepatitis C by product from March 2016 to December 2024.

An average of 4051 prescriptions for HCV DAA were dispensed each month over the study period. Claims processed for dispensings of HCV DAA treatment peaked in June 2016 (20,231 dispensings), 4 months after the initial PBS‐listing in March 2016 (Figure 2). In June 2016, the most commonly dispensed DAAs were ledipasvir with sofosbuvir (7860 dispensings processed, 39% of overall monthly DAA dispensings), sofosbuvir (5967 dispensings processed, 30% of overall monthly DAA dispensings) and daclatasvir (5477 dispensings processed, 27% of overall monthly DAA dispensings).

**FIGURE 3:**
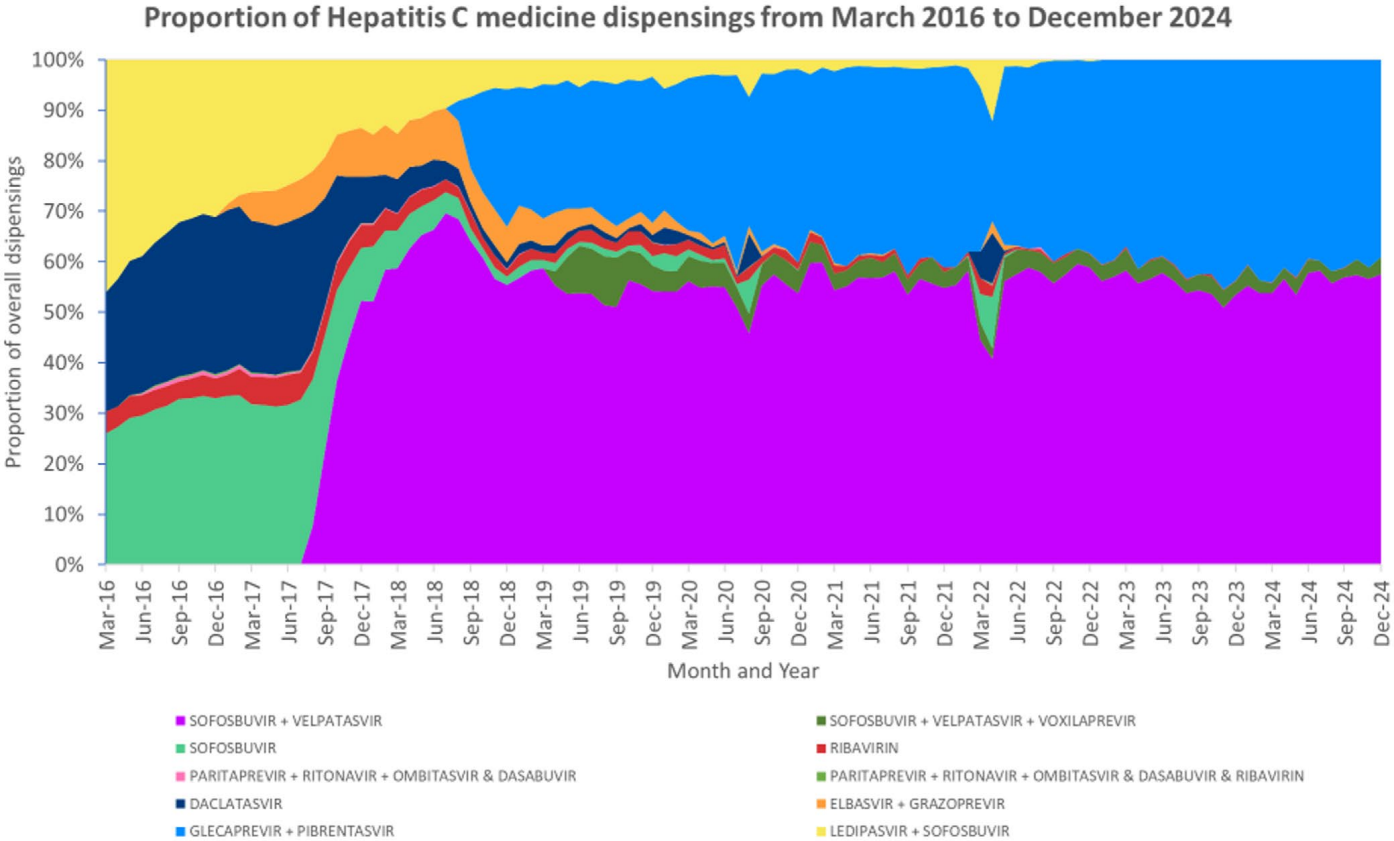
Overall proportion (market share) of direct acting antiviral treatments by product per month.

Since June 2016, monthly dispensings of DAAs have declined. Over 2016, most dispensings of hepatitis C medicines could be attributed to ledipasvir/sofosbuvir, sofosbuvir, or daclatasvir, which accounted for an average of 36%, 31% and 28% of HCV dispensings each month respectively (Figure 3). Ribavirin monthly dispensings initially averaged 4.7% of all monthly dispensings from March 2016 until December 2017 but declined to 0% when delisted in November 2024.

Elbasvir/grazoprevir was listed on the PBS in January 2017, accounting for 10% of all monthly PBS dispensings for HCV medicines in July 2018; however, monthly dispensings declined thereafter aligning with a corresponding increase in sofosbuvir/velpatasvir monthly dispensings (Figure 3).

Since introduction to market in August 2017, the pan‐genotypic regimen sofosbuvir/velpatasvir has maintained an average of 55% of HCV medicine dispensings (Figure 3). The majority of sofosbuvir/velpatasvir dispensings were dispensed via the General Schedule (average of 75% of monthly sofosbuvir/velpatasvir dispensings over the study period) with two repeats allowing for 12 weeks of treatment which is the standard duration of therapy (Figure A2). A smaller proportion of 24% of monthly sofosbuvir/velpatasvir dispensings over the study period were attributable to Public Hospital prescription dispensings with up to two repeats.

For the pan‐genotypic regimen glecaprevir/pibrentasvir, since introduction to market in August 2018 [14], average market share has been 34% (Figure 3). For glecaprevir/pibrentasvir, most of the dispensings were for prescriptions dispensed on either the PBS General Schedule (average of 49% of glecaprevir/pibrentasvir monthly dispensings across study period) or the Public Hospital Schedule (average of 37.5% of glecaprevir/pibrentasvir monthly dispensings across study period), with up to one repeat, equivalent to standard therapy for 8 weeks of treatment (Figure A8).

A smaller average market share of 4% was attributed to the pan‐genotypic ‘salvage therapy’ sofosbuvir/velpatasvir/voxilaprevir since PBS listing in April 2019 (Figure 1, Figure 3). The majority of sofosbuvir/velpatasvir/voxilaprevir dispensing claims were for prescriptions dispensed via the General Schedule with two repeats, equivalent to 12 weeks supply, the standard duration of salvage therapy (Figure A3).

From January 2021, dispensings of DAAs have stabilised at an average of 1442 dispensings per month. The number of dispensings per month and year for each DAA in Australia over the study period by product, strength and number of repeats is included in Figures A1–A10.

## 4 Discussion

Sofosbuvir/velpatasvir and glecaprevir/pibrentasvir have been the most commonly dispensed DAAs since September 2018 in Australia. This distribution of DAA dispensings aligns with current treatment guidelines. Both regimens are pan‐genotypic and effective against all six major hepatitis C virus (HCV) genotypes, making them the preferred first‐line treatments. Prior to September 2018, genotype‐specific DAAs were most commonly dispensed, including ledipasvir/sofosbuvir (subsidised for Genotype 1 and patients with cirrhosis), sofosbuvir, and also daclatasvir, although the latter two were not available in a combination product. When daclatasvir was provided with sofosbuvir, it was subsidised on the PBS for patients with HCV Genotype 1 or 3 and if they had cirrhosis [14]. Additionally, sofosbuvir with ribavirin was subsidised for patients with Genotype 2 or 3 and if they had cirrhosis [14].

These Australian trends are consistent with international observations. Globally, sofosbuvir/velpatasvir and glecaprevir/pibrentasvir are the most commonly used DAAs, reflecting their pan‐genotypic activity, high efficacy (SVR rates > 95%), and widespread registration in over 145 countries [10, 15]. These regimens are recommended by the World Health Organization and have replaced older genotype‐specific treatments such as sofosbuvir + daclatasvir and ledipasvir/sofosbuvir as first‐line therapy in most settings. They are also included on the World Health Organization Model List of Essential Medicines [16].

### 4.1 Public Health Implications

The results of this study complement findings from the Kirby Institute, which monitors hepatitis C infections and treatment uptake in Australia. The Kirby Institute has reported that over 105,000 people have been treated with DAAs between 2016 and 2023, with new treatment initiations declining over time in alignment with treating the prevalent population early after DAAs were added to the PBS in 2016 [17]. The Kirby Institute has, however, observed an increasing need for retreatment, with approximately 10% of all treated individuals requiring additional therapy [17]. Furthermore, treatment by gastroenterologists has decreased from 50% to 19% between 2016 and 2023, while general practitioners now treat more than 55% of patients [17]. Our analysis aligns with these findings, showing that the majority of DAA dispensings occur in the community setting rather than the hospital setting. Receiving hepatitis C care in the community setting rather than the hospital setting is likely to reduce stigma and improve patient access to care, both of which are important factors in person‐centred care and successful hepatitis C treatment [18].

### 4.2 Treatment Duration and Adherence

Treatment duration differs between the two predominant pan‐genotypic regimens being used in practice. Glecaprevir/pibrentasvir is typically administered for eight weeks [19], whereas sofosbuvir/velpatasvir is typically prescribed for 12 weeks [20, 21]. Some patients who use glecaprevir/pibrentasvir may need an extended duration of treatment (12–16 weeks) if they have compensated cirrhosis (Child‐Pugh A) or are treatment‐experienced [20]. However, it is unclear whether patients are receiving or completing the correct duration of treatment. Data from the Kirby Institute's July 2024 report indicate that in 2023, most people received either 8 or 12 weeks of treatment, but the data were not subdivided by specific regimen [17]. Further investigation into treatment duration is necessary to determine whether patients are receiving appropriate therapy; however, an alternative dataset such as the PBS 10% extract would need to be utilised as repeat prescription intervals can be used to infer the course of treatment. Given that approximately 10% of the treated population now requires retreatment [17], ensuring that longer treatment durations of glecaprevir/pibrentasvir are prescribed when indicated is important. Sofosbuvir/velpatasvir/voxilaprevir accounted for only 4% of total monthly hepatitis C DAA dispensings, which is expected given its role primarily as a salvage therapy. This combination is primarily reserved for patients who have experienced virological failure with first‐line therapy, as per therapeutic guidelines [7]. Our study indicated that most DAA prescriptions dispensed were for PBS items that allowed prescription of sufficient repeats for 8–12 weeks of treatment. However, prescribers can choose to prescribe fewer repeats than the maximum number allowed, and so we cannot be certain that all patients received the maximum number of repeats allowing 8–12 weeks of treatment. Further analyses using individual patient‐level data is needed to answer this question.

### 4.3 Comparative Effectiveness of DAAs and Research Gaps

Through use of this present data, we are unable to determine why patients receive glecaprevir/pibrentasvir or sofosbuvir/velpatasvir. A prospective observational study involving 769 patients found that both regimens (glecaprevir/pibrentasvir and sofosbuvir/velpatasvir) demonstrated high treatment completion rates and efficacy within a unique model of care [22]. While baseline characteristics and outcomes were compared, this was a single‐centre study, limiting generalisability. No statistically significant differences in treatment completion rates were observed between therapies. In terms of drug interactions, in the study, the most common drug–drug interactions involved aripiprazole, quetiapine and PPIs, with sofosbuvir/velpatasvir preferred if aripiprazole or quetiapine were being taken and glecaprevir/pibrentasvir if PPIs were being taken. No participants who completed therapy experienced virologic failure or re‐infection [22]. Another multinational study that assessed sustained virologic response at 12 weeks after end of HCV treatment (SVR12) observed 97.8% cure rates (1192/1219 patients) with use of glecaprevir/pibrentasvir and 96.6% cure rates (1584/1640 patients) with sofosbuvir/velpatasvir with or without ribavirin [23]. In vitro studies suggest that glecaprevir may have superior performance against certain HCV genotypes, such as genotype 2b, compared to sofosbuvir/velpatasvir [24]; however, these findings have not been confirmed in clinical settings, and their relevance to patient care remains uncertain. Moreover, there is limited evidence to suggest that sofosbuvir/velpatasvir (with or without ribavirin) is slightly more effective if there is a history of treatment failure in the presence of fibrosis or cirrhosis. Additional national and international studies are required to elucidate comparative effectiveness and address the gaps described above.

A concerning trend identified in the Kirby Institute report is the rising rate of early treatment discontinuation, now occurring in approximately 14% of patients despite the availability of shorter duration pan‐genotypic DAA therapy [17]. The granular monthly data provided in our study shows good treatment uptake and a movement toward public health goals and WHO targets. Together, epidemiological surveillance by the Kirby Institute and pharmacoepidemiological analysis from our study provide complementary perspectives that are essential for achieving HCV elimination goals. The Kirby Institute focuses on HCV incidence, prevalence, and elimination through epidemiological surveillance, tracking population‐level trends and outcomes (including both clinical and health outcomes). On the other hand, our analysis focused on examining PBS dispensing patterns of HCV DAAs, analysing market share of DAAs over time, and tracking changes due to new listings, delistings and clinical preference shifts.

A key strength of this study is the use of comprehensive, publicly available national data encompassing all PBS‐subsidised dispensings of hepatitis C DAAs across Australia. By analysing monthly prescription counts over nearly 9 years, we were able to quantify the evolving market share of DAAs and identify prescribing trends across both community and hospital settings.

Several limitations however should be acknowledged. First, the PBS data used is aggregate and not patient‐level, meaning that we cannot determine individual treatment courses, adherence, or outcomes such as cure. Second, our proxy measure of treatment duration (maximum repeats) assumes full prescribing behaviour, but prescribers may issue fewer repeats than permitted. Third, the use of claim date as a proxy for supply date is a reasonable assumption given real‐time PBS claiming but may still introduce minimal temporal misclassification. Fourth, this dataset does not include private prescriptions or medicines obtained outside the PBS, although the vast majority of HCV treatment in Australia is government‐subsidised.

## 5 Conclusion

Glecaprevir/pibrentasvir and sofosbuvir/velpatasvir are the current preferred and most commonly dispensed DAAs for the treatment of HCV in Australia. These DAAs are primarily prescribed in the community setting and the maximum repeats allocated to each dispensing indicate that most people are prescribed PBS items which allow the standard duration of treatment. Future studies should focus on determining which patients should receive either pan‐genotypic DAA for optimal effectiveness and safety of treatment.

## Conflicts of Interest

Nicole Pratt is a member of the Drug Utilisation Sub Committee (DUSC) of the Pharmaceutical Benefits Advisory Committee (PBAC).

## Supporting information


**Appendix A1.** Appendix.

## Data Availability

The data that support the findings of this study are openly available in Pharmaceutical Benefits Schedule Item Reports at https://medicarestatistics.humanservices.gov.au/VEA0032/SAS.Web/statistics/pbs_item.html.
