# Supplementary material for: Trends in Use of Direct‐Acting Antivirals for Treatment of Hepatitis C Virus Infection in Australia 2016–2024
Source: J Viral Hepat. 2025 Sep 6;32(10):e70082. doi: 10.1111/jvh.70082 (PMC12413624; doi:10.1111/jvh.70082)

**Appendix:**

**Table 1: Pharmaceutical Benefits Scheme item codes, medicine name and strength for each hepatitis C medicine. S100 = Section 100, HSD = highly specialised drug scheme**

| **ATC Code** | **PBS Item code, Medicine name, formulation, strength, dispense setting, number of repeats** |
| --- | --- |
| J05AP01 | 10623D RIBAVIRIN Tablet 400 mg S100 HSD Private 2 repeats |
| J05AP01 | 10635R RIBAVIRIN Tablet 400 mg S100 HSD Private 5 repeats |
| J05AP01 | 10637W RIBAVIRIN Tablet 600 mg S100 HSD Private 5 repeats |
| J05AP01 | 10638X RIBAVIRIN Tablet 600 mg S100 HSD Public 5 repeats |
| J05AP01 | 10646H RIBAVIRIN Tablet 400 mg S100 HSD Public 5 repeats |
| J05AP01 | 10647J RIBAVIRIN Tablet 400 mg General schedule 2 repeats |
| J05AP01 | 10663F RIBAVIRIN Tablet 600 mg S100 HSD Public 2 repeats |
| J05AP01 | 10665H RIBAVIRIN Tablet 600 mg General schedule 2 repeats |
| J05AP01 | 10666J RIBAVIRIN Tablet 600 mg General schedule 5 repeats |
| J05AP01 | 10673R RIBAVIRIN Tablet 400 mg General schedule 5 repeats |
| J05AP01 | 10675W RIBAVIRIN Tablet 600 mg S100 HSD Private 2 repeats |
| J05AP01 | 10678B RIBAVIRIN Tablet 400 mg S100 HSD Public 2 repeats |
| J05AP01 | 10914K RIBAVIRIN Tablet 200 mg S100 HSD Public 5 repeats |
| J05AP01 | 10923X RIBAVIRIN Tablet 200 mg S100 HSD Private 2 repeats |
| J05AP01 | 10928E RIBAVIRIN Tablet 200 mg General schedule 5 repeats |
| J05AP01 | 10929F RIBAVIRIN Tablet 200 mg S100 HSD Public 2 repeats |
| J05AP01 | 10937P RIBAVIRIN Tablet 200 mg General schedule 2 repeats |
| J05AP01 | 10938Q RIBAVIRIN Tablet 200 mg S100 HSD Private 5 repeats |
| J05AP01 | 12785X RIBAVIRIN Tablet 200 mg General schedule 2 repeats |
| J05AP01 | 12786Y RIBAVIRIN Tablet 200 mg S100 HSD Public 2 repeats |
| J05AP01 | 12809E RIBAVIRIN Tablet 200 mg S100 HSD Private 2 repeats |
| J05AP07 | 10629K DACLATASVIR Tablet 30 mg S100 HSD Public 5 repeats |
| J05AP07 | 10630L DACLATASVIR Tablet 30 mg S100 HSD Private 5 repeats |
| J05AP07 | 10631M DACLATASVIR Tablet 60 mg S100 HSD Private 5 repeats |
| J05AP07 | 10641C DACLATASVIR Tablet 60 mg S100 HSD Public 5 repeats |
| J05AP07 | 10642D DACLATASVIR Tablet 60 mg General schedule 2 repeats |
| J05AP07 | 10643E DACLATASVIR Tablet 30 mg S100 HSD Private 2 repeats |
| J05AP07 | 10644F DACLATASVIR Tablet 60 mg S100 HSD Private 2 repeats |
| J05AP07 | 10645G DACLATASVIR Tablet 30 mg General schedule 2 repeats |
| J05AP07 | 10651N DACLATASVIR Tablet 30 mg S100 HSD Public 2 repeats |
| J05AP07 | 10659B DACLATASVIR Tablet 60 mg General schedule 5 repeats |
| J05AP07 | 10660C DACLATASVIR Tablet 60 mg S100 HSD Public 2 repeats |
| J05AP07 | 10671P DACLATASVIR Tablet 30 mg General schedule 5 repeats |
| J05AP08 | 10624E SOFOSBUVIR Tablet 400 mg General schedule 1 repeat |
| J05AP08 | 10625F SOFOSBUVIR Tablet 400 mg S100 HSD Public 2 repeats |
| J05AP08 | 10648K SOFOSBUVIR Tablet 400 mg S100 HSD Public 5 repeats |
| J05AP08 | 10654R SOFOSBUVIR Tablet 400 mg S100 HSD Private 2 repeats |
| J05AP08 | 10657X SOFOSBUVIR Tablet 400 mg General schedule 5 repeats |
| J05AP08 | 10676X SOFOSBUVIR Tablet 400 mg S100 HSD Private 5 repeats |
| J05AP51 | 10628J LEDIPASVIR + SOFOSBUVIR Tablet containing 90 mg ledipasvir with 400 mg sofosbuvir General schedule 2 repeats |
| J05AP51 | 10653Q LEDIPASVIR + SOFOSBUVIR Tablet containing 90 mg ledipasvir with 400 mg sofosbuvir S100 HSD Private 1 repeat |
| J05AP51 | 10661D LEDIPASVIR + SOFOSBUVIR Tablet containing 90 mg ledipasvir with 400 mg sofosbuvir S100 HSD Public 2 repeats |
| J05AP51 | 10667K LEDIPASVIR + SOFOSBUVIR Tablet containing 90 mg ledipasvir with 400 mg sofosbuvir S100 HSD Public 1 repeat |
| J05AP51 | 10668L LEDIPASVIR + SOFOSBUVIR Tablet containing 90 mg ledipasvir with 400 mg sofosbuvir General schedule 1 repeat |
| J05AP51 | 10669M LEDIPASVIR + SOFOSBUVIR Tablet containing 90 mg ledipasvir with 400 mg sofosbuvir S100 HSD Public 5 repeats |
| J05AP51 | 10670N LEDIPASVIR + SOFOSBUVIR Tablet containing 90 mg ledipasvir with 400 mg sofosbuvir General schedule 5 repeats |
| J05AP51 | 10672Q LEDIPASVIR + SOFOSBUVIR Tablet containing 90 mg ledipasvir with 400 mg sofosbuvir S100 HSD Private 2 repeats |
| J05AP51 | 10679C LEDIPASVIR + SOFOSBUVIR Tablet containing 90 mg ledipasvir with 400 mg sofosbuvir S100 HSD Private 5 repeats |
| J05AP52 | 10747P PARITAPREVIR + RITONAVIR + OMBITASVIR & DASABUVIR & RIBAVIRIN Pack containing 56 tablets paritaprevir 75 mg with ritonavir 50 mg with ombitasvir 12.5 mg and 56 tablets dasabuvir 250 mg and 56 tablets ribavirin 600 mg General schedule 5 repeats |
| J05AP52 | 10749R PARITAPREVIR + RITONAVIR + OMBITASVIR & DASABUVIR Pack containing 56 tablets paritaprevir 75 mg with ritonavir 50 mg with ombitasvir 12.5 mg and 56 tablets dasabuvir 250 mg S100 HSD Private 2 repeats |
| J05AP52 | 10750T PARITAPREVIR + RITONAVIR + OMBITASVIR & DASABUVIR & RIBAVIRIN Pack containing 56 tablets paritaprevir 75 mg with ritonavir 50 mg with ombitasvir 12.5 mg and 56 tablets dasabuvir 250 mg and 56 tablets ribavirin 600 mg S100 HSD Private 2 repeats |
| J05AP52 | 10751W PARITAPREVIR + RITONAVIR + OMBITASVIR & DASABUVIR Pack containing 56 tablets paritaprevir 75 mg with ritonavir 50 mg with ombitasvir 12.5 mg and 56 tablets dasabuvir 250 mg S100 HSD Public 2 repeats |
| J05AP52 | 10752X PARITAPREVIR + RITONAVIR + OMBITASVIR & DASABUVIR & RIBAVIRIN Pack containing 56 tablets paritaprevir 75 mg with ritonavir 50 mg with ombitasvir 12.5 mg and 56 tablets dasabuvir 250 mg and 168 tablets ribavirin 200 mg S100 HSD Public 5 repeats |
| J05AP52 | 10753Y PARITAPREVIR + RITONAVIR + OMBITASVIR & DASABUVIR & RIBAVIRIN Pack containing 56 tablets paritaprevir 75 mg with ritonavir 50 mg with ombitasvir 12.5 mg and 56 tablets dasabuvir 250 mg and 168 tablets ribavirin 200 mg S100 HSD Private 2 repeats |
| J05AP52 | 10754B PARITAPREVIR + RITONAVIR + OMBITASVIR & DASABUVIR & RIBAVIRIN Pack containing 56 tablets paritaprevir 75 mg with ritonavir 50 mg with ombitasvir 12.5 mg and 56 tablets dasabuvir 250 mg and 56 tablets ribavirin 600 mg S100 HSD Public 2 repeats |
| J05AP52 | 10761J PARITAPREVIR + RITONAVIR + OMBITASVIR & DASABUVIR & RIBAVIRIN Pack containing 56 tablets paritaprevir 75 mg with ritonavir 50 mg with ombitasvir 12.5 mg and 56 tablets dasabuvir 250 mg and 168 tablets ribavirin 200 mg S100 HSD Private 5 repeats |
| J05AP52 | 10765N PARITAPREVIR + RITONAVIR + OMBITASVIR & DASABUVIR & RIBAVIRIN Pack containing 56 tablets paritaprevir 75 mg with ritonavir 50 mg with ombitasvir 12.5 mg and 56 tablets dasabuvir 250 mg and 168 tablets ribavirin 200 mg S100 HSD Public 2 repeats |
| J05AP52 | 10766P PARITAPREVIR + RITONAVIR + OMBITASVIR & DASABUVIR Pack containing 56 tablets paritaprevir 75 mg with ritonavir 50 mg with ombitasvir 12.5 mg and 56 tablets dasabuvir 250 mg General schedule 2 repeats |
| J05AP52 | 10768R PARITAPREVIR + RITONAVIR + OMBITASVIR & DASABUVIR & RIBAVIRIN Pack containing 56 tablets paritaprevir 75 mg with ritonavir 50 mg with ombitasvir 12.5 mg and 56 tablets dasabuvir 250 mg and 56 tablets ribavirin 600 mg S100 HSD Public 5 repeats |
| J05AP52 | 10769T PARITAPREVIR + RITONAVIR + OMBITASVIR & DASABUVIR & RIBAVIRIN Pack containing 56 tablets paritaprevir 75 mg with ritonavir 50 mg with ombitasvir 12.5 mg and 56 tablets dasabuvir 250 mg and 56 tablets ribavirin 600 mg General schedule 2 repeats |
| J05AP52 | 10771X PARITAPREVIR + RITONAVIR + OMBITASVIR & DASABUVIR & RIBAVIRIN Pack containing 56 tablets paritaprevir 75 mg with ritonavir 50 mg with ombitasvir 12.5 mg and 56 tablets dasabuvir 250 mg and 168 tablets ribavirin 200 mg General schedule 5 repeats |
| J05AP52 | 10772Y PARITAPREVIR + RITONAVIR + OMBITASVIR & DASABUVIR & RIBAVIRIN Pack containing 56 tablets paritaprevir 75 mg with ritonavir 50 mg with ombitasvir 12.5 mg and 56 tablets dasabuvir 250 mg and 168 tablets ribavirin 200 mg General schedule 2 repeats |
| J05AP52 | 10773B PARITAPREVIR + RITONAVIR + OMBITASVIR & DASABUVIR & RIBAVIRIN Pack containing 56 tablets paritaprevir 75 mg with ritonavir 50 mg with ombitasvir 12.5 mg and 56 tablets dasabuvir 250 mg and 56 tablets ribavirin 600 mg S100 HSD Private 5 repeats |
| J05AP54 | 10978T ELBASVIR + GRAZOPREVIR Tablet containing grazoprevir 100 mg with elbasvir 50 mg S100 HSD Public 2 repeats |
| J05AP54 | 10979W ELBASVIR + GRAZOPREVIR Tablet containing grazoprevir 100 mg with elbasvir 50 mg S100 HSD Private 2 repeats |
| J05AP54 | 10986F ELBASVIR + GRAZOPREVIR Tablet containing grazoprevir 100 mg with elbasvir 50 mg S100 HSD Public 3 repeats |
| J05AP54 | 10991L ELBASVIR + GRAZOPREVIR Tablet containing grazoprevir 100 mg with elbasvir 50 mg S100 HSD Private 3 repeats |
| J05AP54 | 11011M ELBASVIR + GRAZOPREVIR Tablet containing grazoprevir 100 mg with elbasvir 50 mg General schedule 3 repeats |
| J05AP54 | 11021C ELBASVIR + GRAZOPREVIR Tablet containing grazoprevir 100 mg with elbasvir 50 mg General schedule 2 repeats |
| J05AP55 | 11144M SOFOSBUVIR + VELPATASVIR Tablet containing 400 mg sofosbuvir with 100 mg velpatasvir S100 HSD Private 2 repeats |
| J05AP55 | 11145N SOFOSBUVIR + VELPATASVIR Tablet containing 400 mg sofosbuvir with 100 mg velpatasvir S100 HSD Public 2 repeats |
| J05AP55 | 11147Q SOFOSBUVIR + VELPATASVIR Tablet containing 400 mg sofosbuvir with 100 mg velpatasvir General schedule 2 repeats |
| J05AP56 | 11658N SOFOSBUVIR + VELPATASVIR + VOXILAPREVIR Tablet containing 400 mg sofosbuvir with 100 mg velpatasvir and 100 mg voxilaprevir General schedule 2 repeats |
| J05AP56 | 11659P SOFOSBUVIR + VELPATASVIR + VOXILAPREVIR Tablet containing 400 mg sofosbuvir with 100 mg velpatasvir and 100 mg voxilaprevir S100 HSD Private 2 repeats |
| J05AP56 | 11665Y SOFOSBUVIR + VELPATASVIR + VOXILAPREVIR Tablet containing 400 mg sofosbuvir with 100 mg velpatasvir and 100 mg voxilaprevir S100 HSD Public 2 repeats |
| J05AP57 | 11332K GLECAPREVIR + PIBRENTASVIR Tablet containing 100 mg glecaprevir with 40 mg pibrentasvir S100 HSD Public 1 repeat |
| J05AP57 | 11333L GLECAPREVIR + PIBRENTASVIR Tablet containing 100 mg glecaprevir with 40 mg pibrentasvir S100 HSD Public 3 repeats |
| J05AP57 | 11337Q GLECAPREVIR + PIBRENTASVIR Tablet containing 100 mg glecaprevir with 40 mg pibrentasvir S100 HSD Private 3 repeats |
| J05AP57 | 11344C GLECAPREVIR + PIBRENTASVIR Tablet containing 100 mg glecaprevir with 40 mg pibrentasvir General Schedule 3 repeats |
| J05AP57 | 11345D GLECAPREVIR + PIBRENTASVIR Tablet containing 100 mg glecaprevir with 40 mg pibrentasvir S100 HSD Public 2 repeats |
| J05AP57 | 11346E GLECAPREVIR + PIBRENTASVIR Tablet containing 100 mg glecaprevir with 40 mg pibrentasvir S100 HSD Private 2 repeats |
| J05AP57 | 11353M GLECAPREVIR + PIBRENTASVIR Tablet containing 100 mg glecaprevir with 40 mg pibrentasvir General schedule 1 repeat |
| J05AP57 | 11354N GLECAPREVIR + PIBRENTASVIR Tablet containing 100 mg glecaprevir with 40 mg pibrentasvir General schedule 2 repeats |
| J05AP57 | 11355P GLECAPREVIR + PIBRENTASVIR Tablet containing 100 mg glecaprevir with 40 mg pibrentasvir S100 HSD Private 1 repeat |

Appendix Table 2: medicine shortages archived reports from TGA website: deletions of products from market.

| **Product name and brand** | **Deletion from market** | **Shortage impact rating** | **Reason** |
| --- | --- | --- | --- |
| IBAVYR ribavirin 400 mg tablet bottle | 1/09/2021 | Low | Commercial Changes / Commercial viability |
| IBAVYR ribavirin 600 mg tablet bottle | 29/10/2021 | Low | Commercial Changes / Commercial viability |
| DAKLINZA daclatasvir (as dihydrochloride) 60 mg tablet blister | 1/10/2020 | Low | Commercial Changes / Commercial viability |
| DAKLINZA daclatasvir (as dihydrochloride) 30 mg tablet blister | 1/10/2020 | Low | Commercial Changes / Commercial viability |
| IBAVYR ribavirin 200 mg film coated tablets bottle | 3/06/2019 | Low | Commercial Changes / Commercial viability |
| ZEPATIER elbasvir / grazoprevir 50mg / 100mg tablet blister pack | 1/04/2021 | Low | Commercial Changes / Commercial viability |

Appendix Figure 1: Sofosbuvir with velpatasvir dispensings processed between March2016 and December 2024 by PBS item code and dispensing setting.


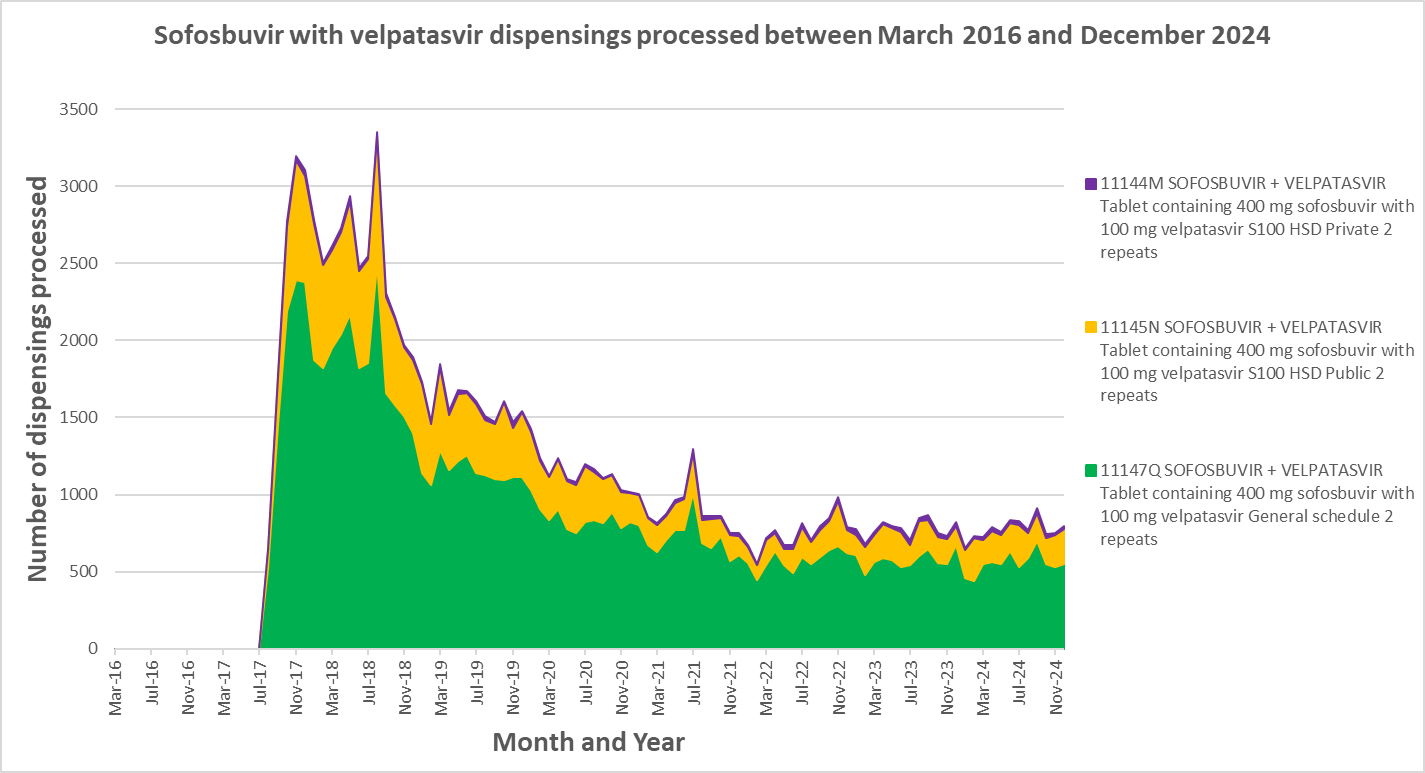


Appendix Figure 2: Sofosbuvir with velpatasvir and voxilaprevir dispensings processed between March 2016 and December 2024 by PBS item code and dispensing setting.


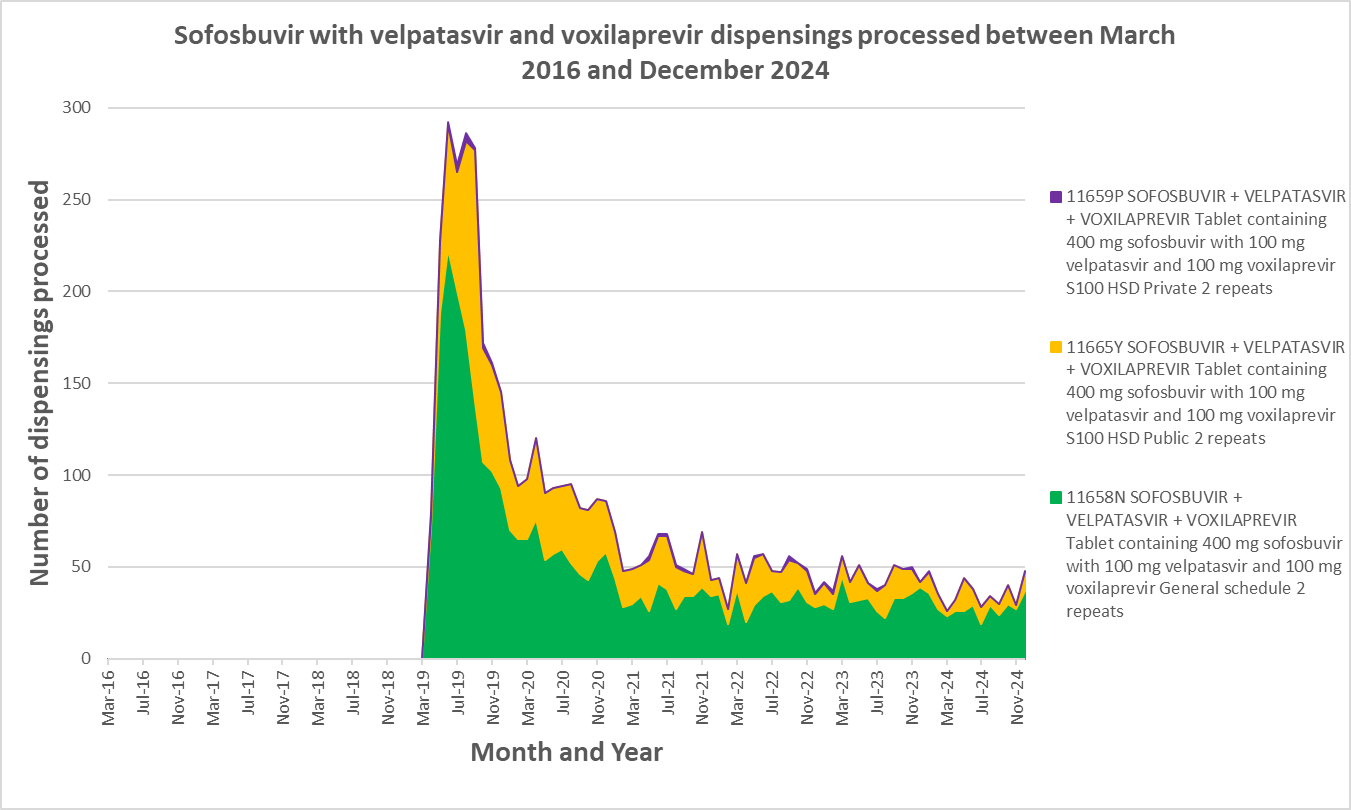


Appendix Figure 3: Sofosbuvir dispensings processed between March 2016 and December 2024 by PBS item code and dispensing setting.


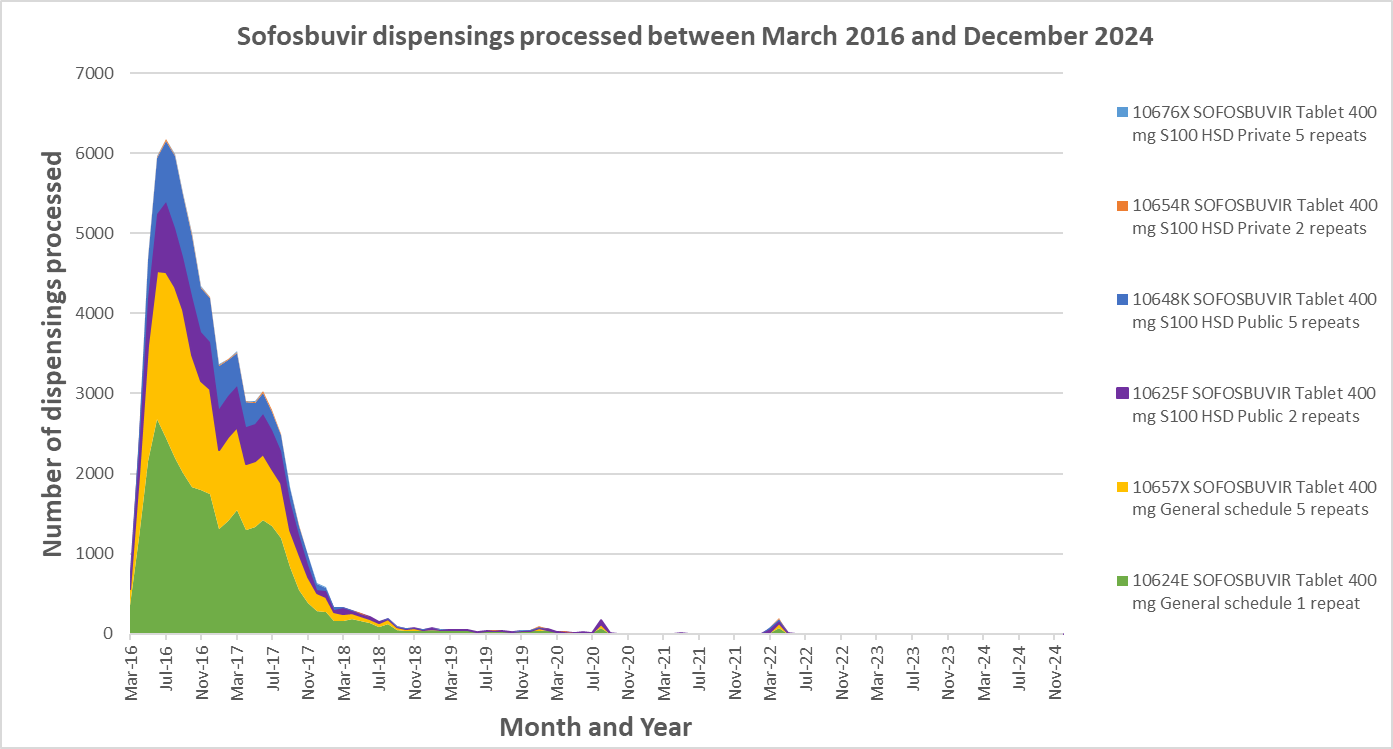


Appendix Figure 4: Ribavirin dispensings processed between March 2016 and December 2024 by PBS item code and dispensing setting.


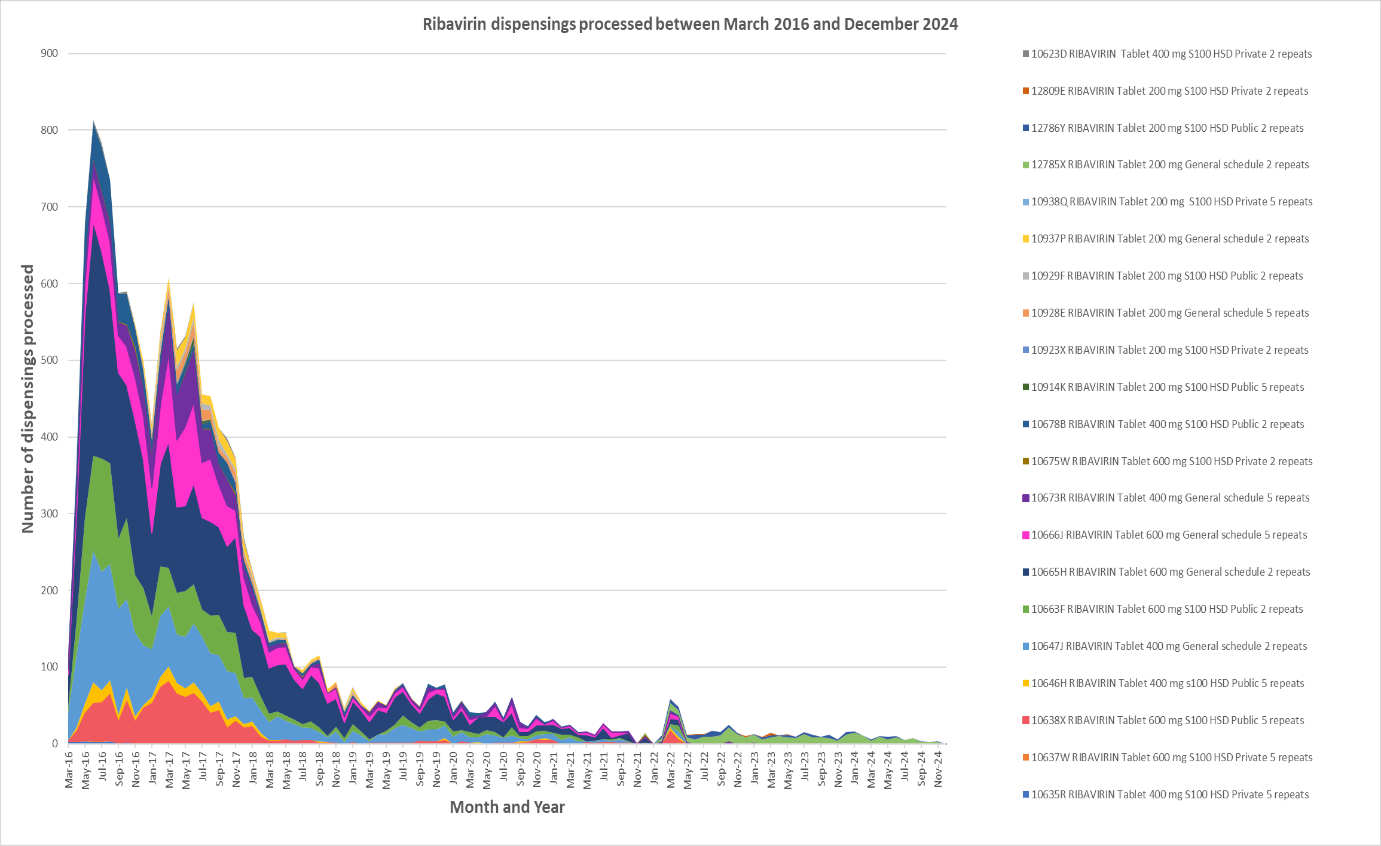


Appendix Figure 5: Paritaprevir with ritonavir, ombitasvir, dasabuvir and ribavirin dispensings processed between March 2016 and December 2024 by PBS item code and dispensing setting.


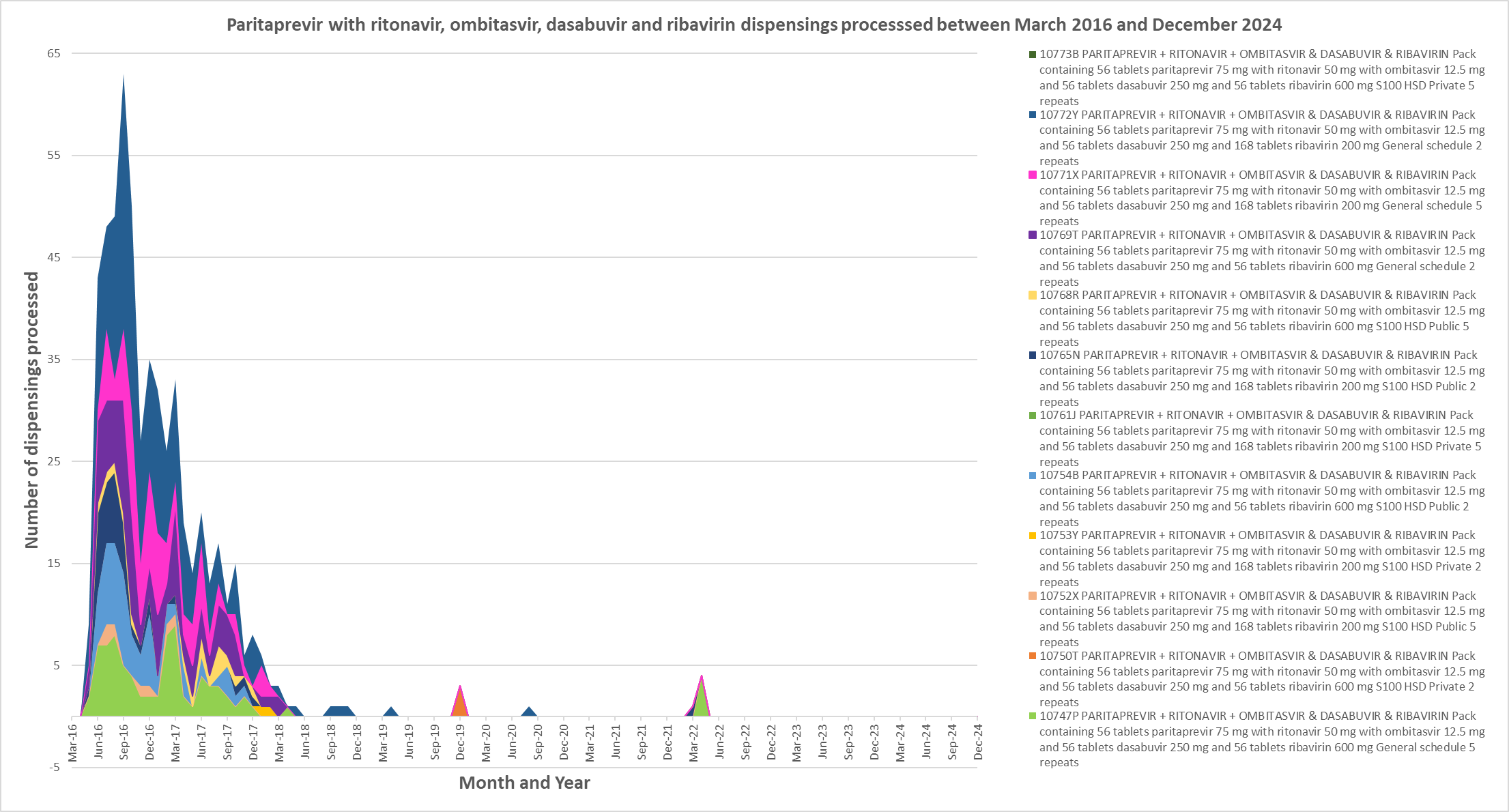


Appendix Figure 6: Daclatasvir dispensings processed between March 2016 and December 2024 by PBS item code and dispensing setting.


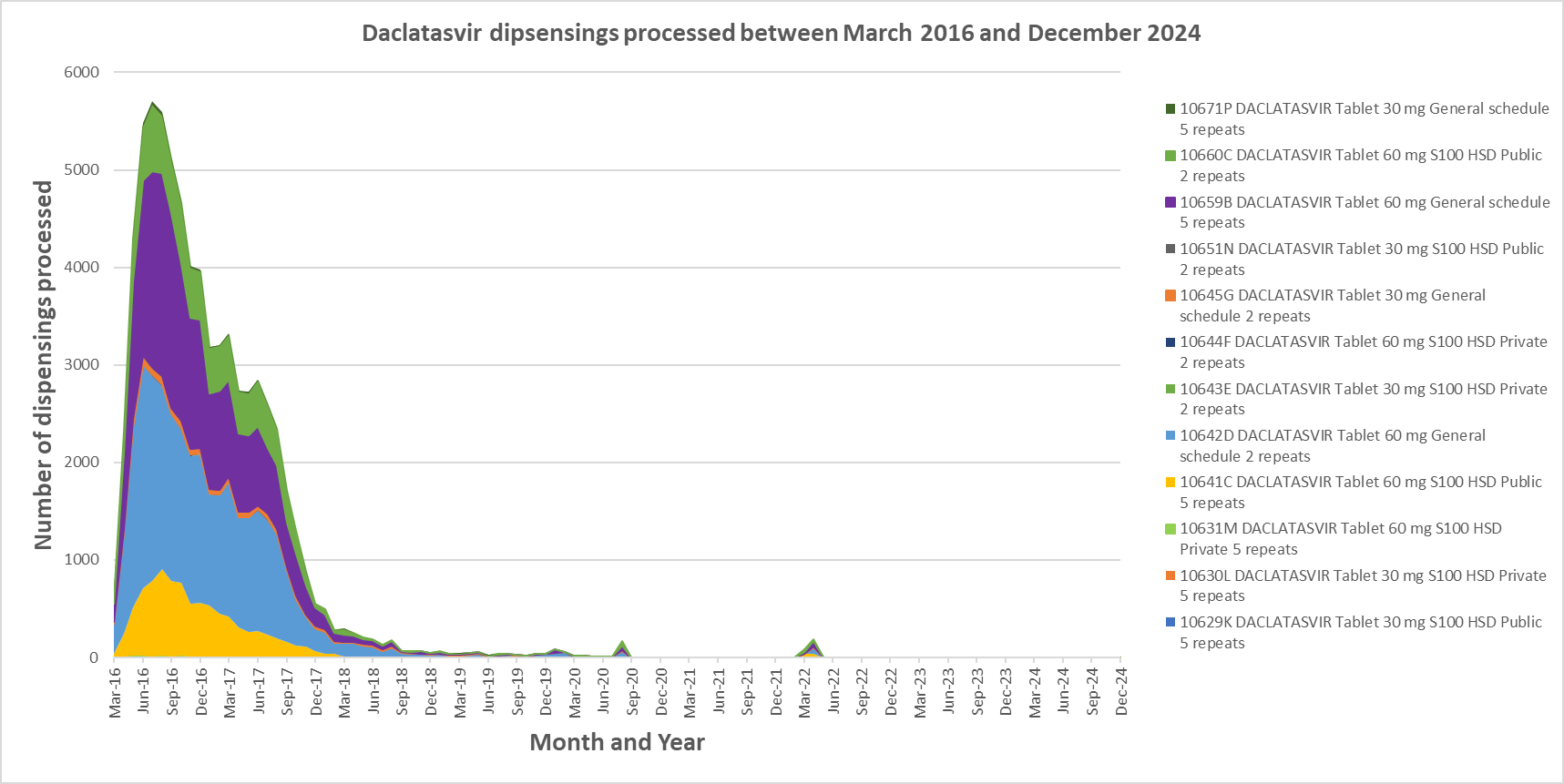


Appendix Figure 7: Elbasvir with grazoprevir dispensings processed between March 2016 and December 2024 by PBS item code and dispensing setting.


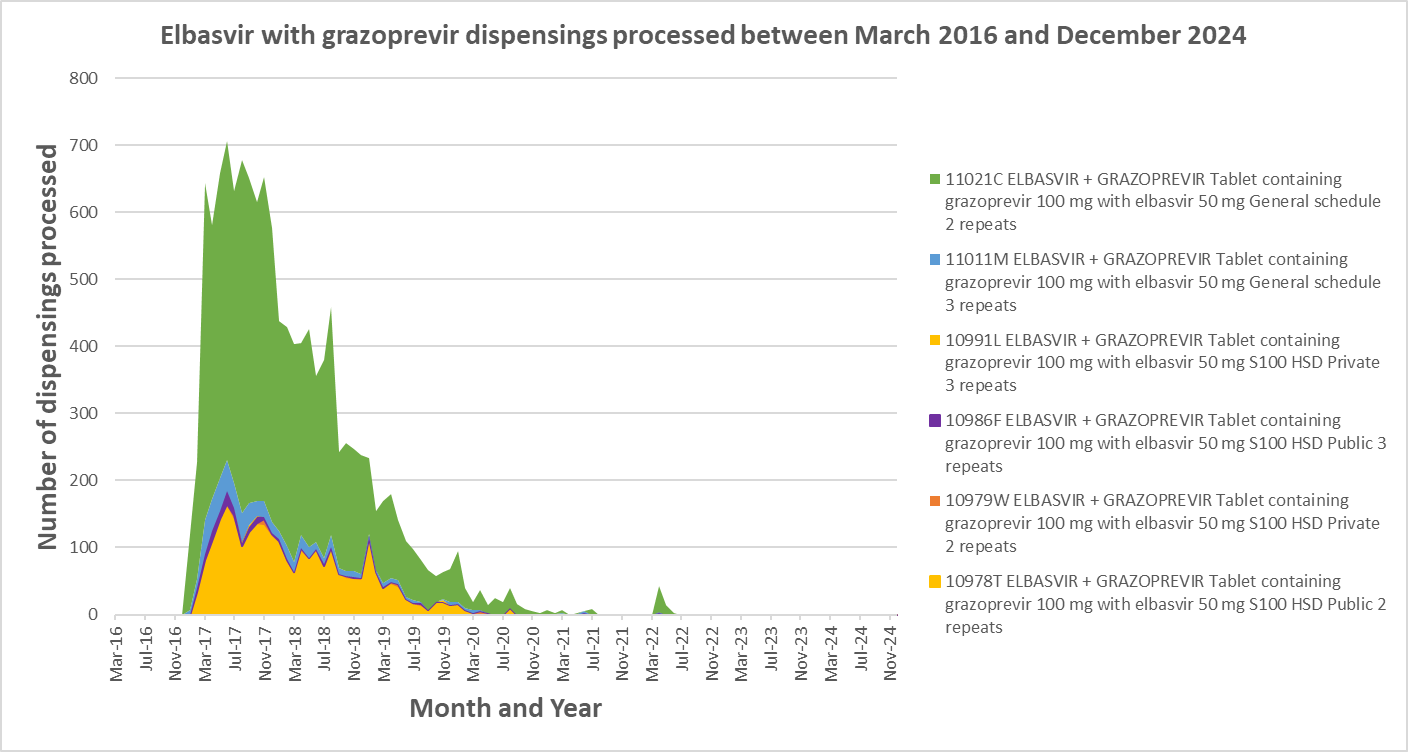


Appendix Figure 8: Glecapevir with pibrentasvir dispensings processed between March 2016 and December 2024 by PBS item code and dispensing setting.


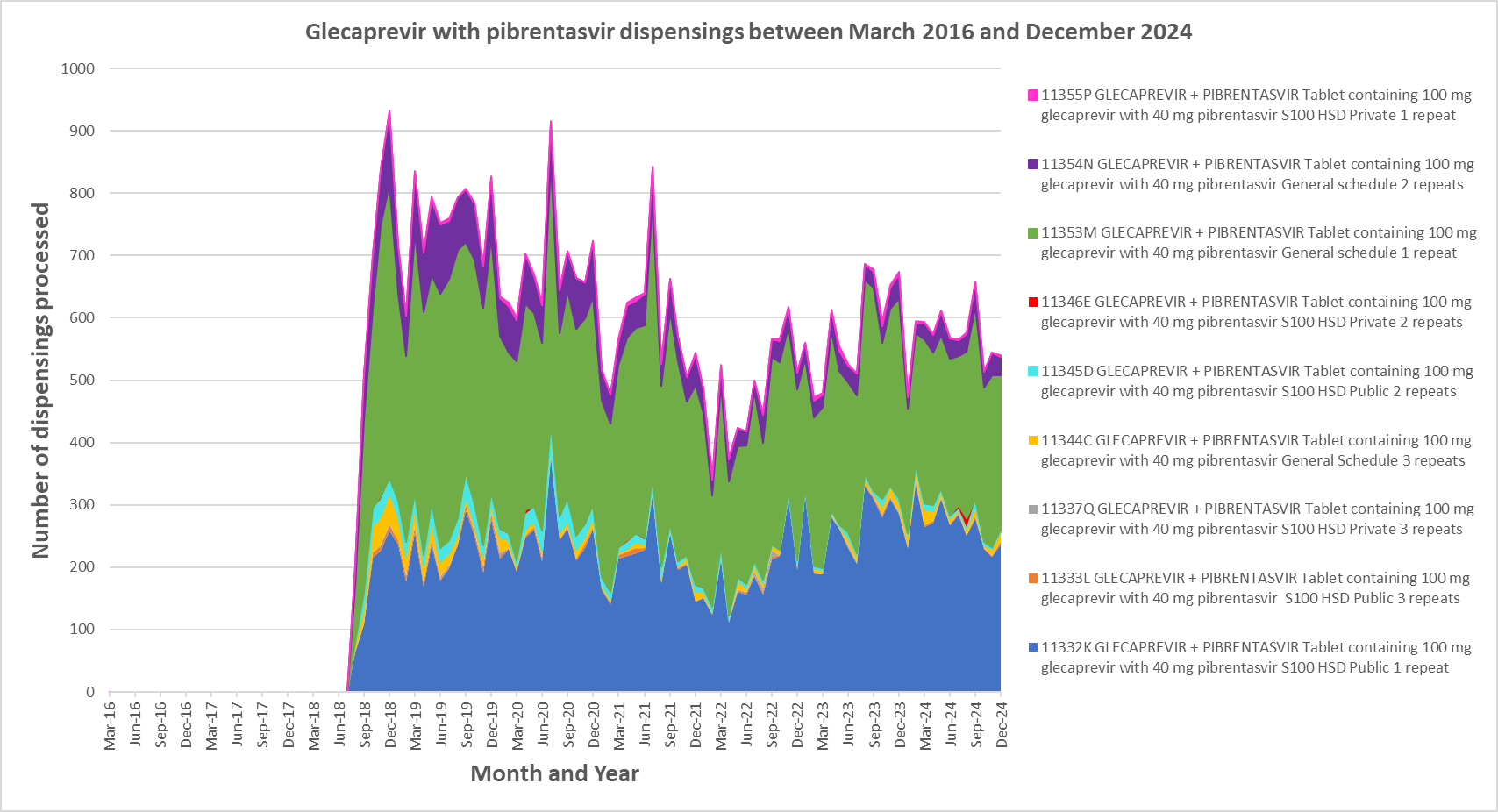


Appendix Figure 9: Ledipasvir with sofosbuvir dispensings processed between March 2016 and December 2024 by PBS item code and dispensing setting.


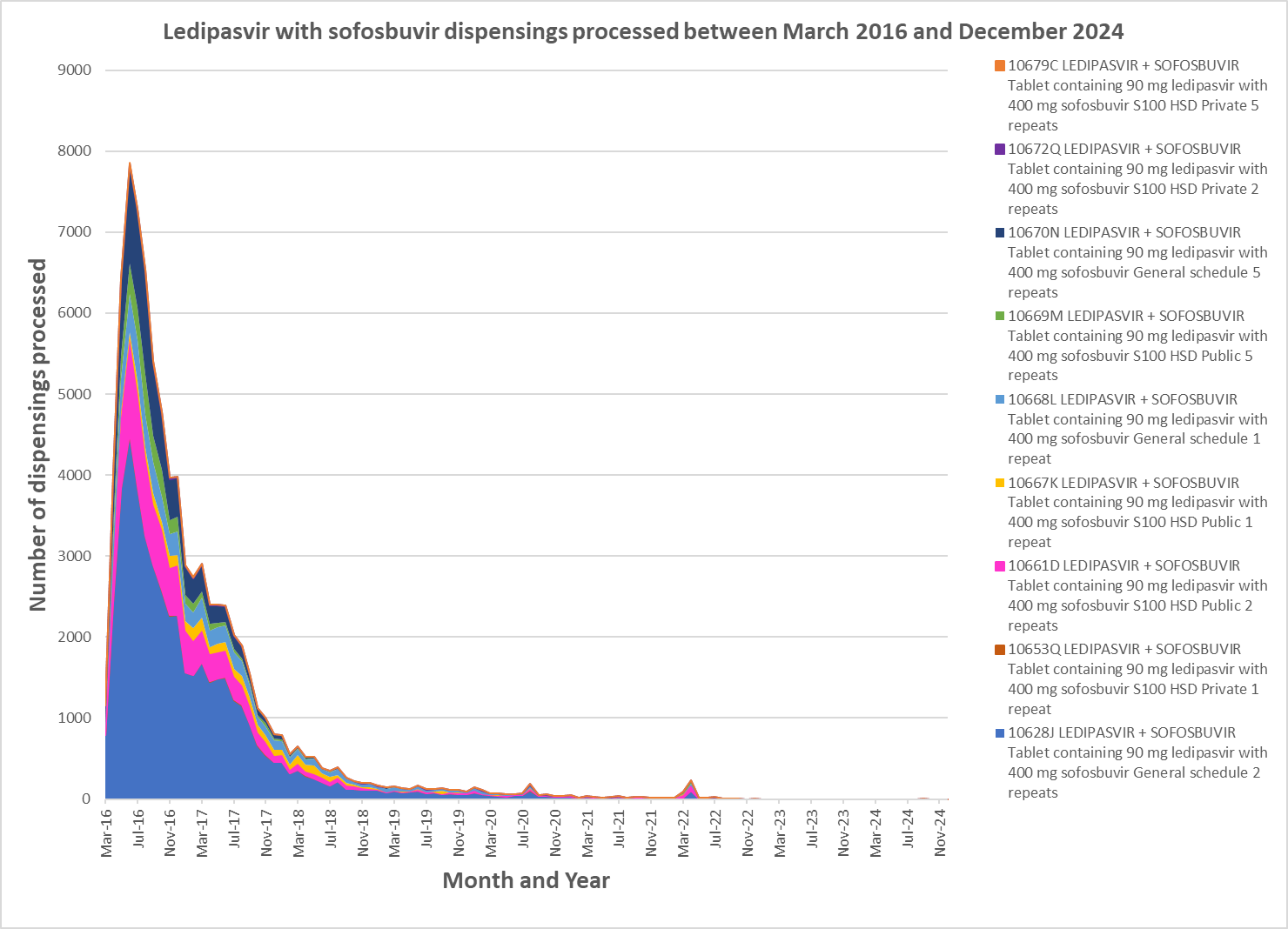


Appendix Figure 10: Paritaprevir with ritonavir, ombitasvir and dasabuvir dispensings processed between March 2016 and December 2024 by PBS item code and dispensing setting.


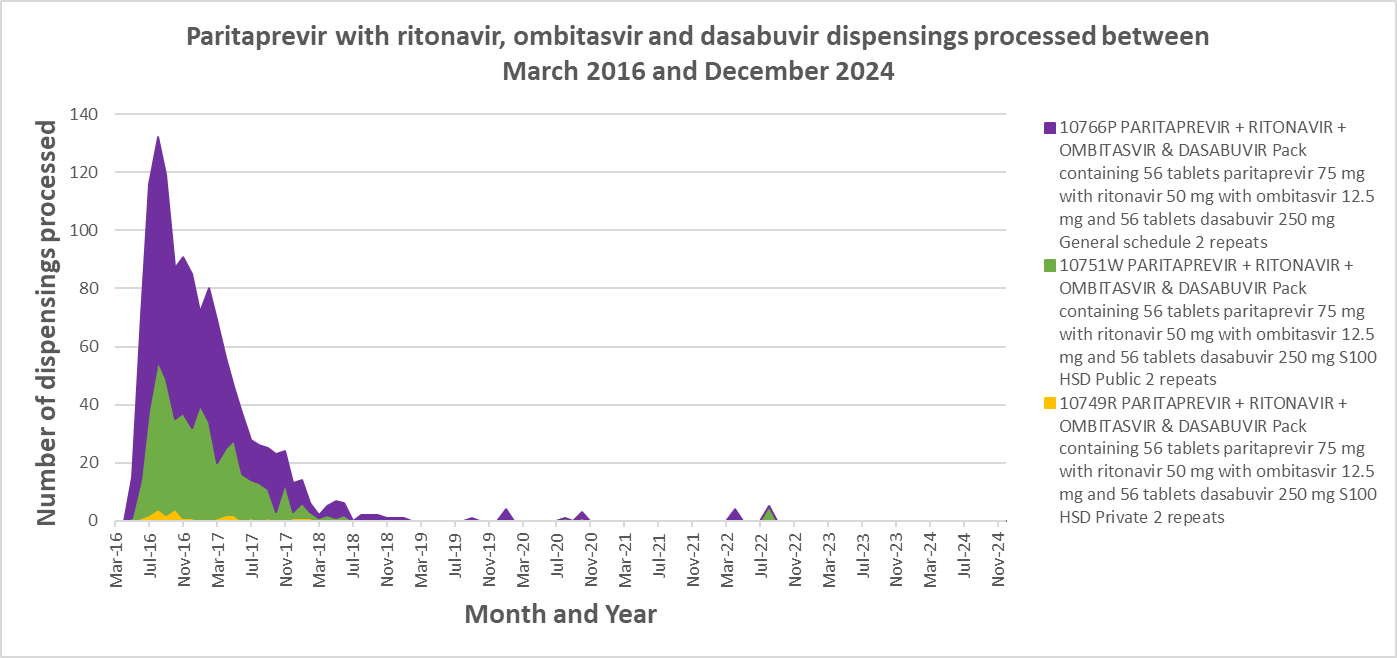

Supplement: Supplementary file 1 — Appendix A1. Appendix. [file JVH-32-0-s001.docx]
